# Exploring the role of socioeconomic factors in the development and spread of anti-malarial drug resistance: a qualitative study

**DOI:** 10.1186/s12936-017-1849-1

**Published:** 2017-05-18

**Authors:** Philip Emeka Anyanwu, John Fulton, Etta Evans, Timothy Paget

**Affiliations:** University of Sunderland, Pasteur Building, Sciences Complex, City Campus, Sunderland, SR1 3SD UK

**Keywords:** Malaria, Drug resistance, Socioeconomic factors, Drug use, Treatment seeking, Behaviour, Antimalarial drugs, Education

## Abstract

**Background:**

Malaria remains a global health issue with the burden unevenly distributed to the disadvantage of the developing countries of the world. Poverty contributes to the malaria burden as it has the ability to affect integral aspects of malaria control. There have been renewed efforts in the global malaria control, resulting in reductions in the global malaria burden over the last decade. However, the development of resistance to artemisinin-based combination therapy threatens the sustainability of the present success in malaria control. Anti-malarial drug use practices/behaviours remain very important drivers of drug resistance. This study adopted a social epidemiological stance in exploring the underlying socioeconomic factors that determine drug use behaviours promoting anti-malarial drug resistance.

**Methods:**

A qualitative approach, involving the use of interviews, was used in this inquiry to explore the existing anti-malarial drug use practices in the Nigerian population; and the different socioeconomic factors influencing the behaviours.

**Results:**

The significant malaria treatment behaviours influenced by socioeconomic factors in this study were the practice of ‘mixing’ drugs for malaria treatment, presumptive treatment, sharing of malaria treatment course, and the use of anti-malaria monotherapies. All the rural dwellers in this study reported they have mixed drugs for malaria treatment. When symptoms were experienced, socio-economic factors, like type of settlement, income level and occupation, tended to determine the treatment behaviour and, therefore, informed and determined the experience of the illness.

**Discussion:**

Social and economic contexts can influence behaviours as they contribute in shaping norms and in creating opportunities that promote certain behaviours. As shown in this study, income level and type of settlement, as structural factors, affect the decision on where to seek malaria treatment and whether or not a malaria diagnostic test will be used prior to treatment. One of the dangers of using the mixed anti-malarial drugs is that it offers a safe route for the sale of expired and fake anti-malarial drugs as the mixed drugs are not sold or dispensed in their original packets.

**Conclusions and recommendations:**

Population-wide improvements in income, education, environmental and structural conditions of rural dwellers in malaria-endemic settings will encourage behavioural change on how anti-malarial drugs are used.

## Background

Malaria is a parasitic infection caused by species of *Plasmodium* parasites [1]. Although a global health issue, its burden is unevenly distributed to the disadvantage of the developing countries of the world [2, 3]. Africa is still the leading region in terms of malaria burden. This region accounts for majority (80%) of the malaria cases as well as malaria-associated deaths (90%) globally [1]; with children under 5 years of age at most risk of malaria infection. Nigeria, a middle-income country in sub-Saharan Africa, is among the countries with the highest malaria burden in the world, with 32% of global malaria mortality occurring in this country and 97% of the population at risk of malaria infection [4]. Some factors that drive the persistence of malaria in the Nigerian population are: the availability of conducive environment for vector breeding [5]; the Nigerian healthcare system; and the high level of poverty in Nigeria [6], where about 46% of the population live below the World Bank poverty line of $1.25 per day [7].

Some features of the Nigerian healthcare system that contribute to the persistence of the disease burden in this population (by constituting a major barrier to early and effective treatment) include the lack of universal and equitable access to primary healthcare as this is the level where conditions like malaria infection are managed [8]; and the current government policy of payment for healthcare in all public health facilities at the point of use [8, 9]. It is established that socioeconomic factors are key drivers of health disparities between and within countries. As a socioeconomic issue, the high level of poverty in Nigeria, as with most malaria endemic countries, is an important factor that reinforces the persistent malaria burden in Nigeria.

Indeed, a strong relationship exists between poverty and malaria [10]. This relationship is evident in the fact that most malaria endemic countries are also among the poorest countries of the world. Poverty contributes to the malaria burden as it has the ability to affect integral aspects of malaria treatment-seeking behaviours [10], including access to preventive measures and treatment—in relation to affordability, acceptability and availability—[11], and adherence to treatment.

Presently, there have been renewed efforts in the global malaria control with several organizations and non-endemic countries increasingly getting involved in the fight against malaria [12, 13]. These efforts have resulted in reduction in the global malaria burden over the last decade [14]. The current achievement is mostly attributed to the increase in malaria research funding, and scale-up of interventions against malaria, including insecticides-treated nets (ITNs), indoor residual spraying (IRS), rapid diagnostic testing (RDT), and importantly, the use of artemisinin-based combination therapy (ACT) [15]. There has been significant improvement in access, availability and affordability of ACT in malaria endemic regions [14].

ACT is presently the World Health Organization (WHO) recommended first-line treatment for uncomplicated malaria [1]. This recommendation has been adopted by most malaria-endemic countries, like Nigeria, where artemether-lumefantrine is the first-line treatment for uncomplicated malaria cases [4, 16]. In relation to previously used anti-malarial drugs, artemisinin drugs are very effective in parasite clearance and can relief the malaria symptoms faster [17, 18]. However, the development of resistance to ACT (which has been confirmed in five countries of Southeast Asia (SEA): Cambodia, Lao PDR, Myanmar, Thailand and Viet Nam [1, 19, 20] poses a major threat to its efficacy and use as first-line treatment. This also threatens the sustainability of the present success in malaria control [21].

Similar to the artemisinin class drugs, the development of resistance to most of the previously used anti-malarial drugs (such as chloroquine, sulfadoxine-pyrimethamine, mefloquine) originated from South East Asia (SEA). Nevertheless, the burden and effects of resistance are usually borne more by the African region that accounts for most of the global malaria cases [1]. Considering the quick and widespread of previous cases of resistance to anti-malarial drugs from the SEA to Africa, the African region stands at risk of spread of artemisinin resistance [22, 23].

Importantly, the mechanism behind the development and spread of anti-malarial drug resistance is a complex one with multiple factors in play—such as drug use practices/behaviours, drug half-life, transmission intensity, clone multiplicity, parasite density, host immunity, within-host dynamics and the genetic basis of drug resistance [24]. Nevertheless, anti-malarial drug use practices/behaviours of presumptive treatment, drug overuse, use of sub-therapeutic doses, non-adherence to the treatment regime, among others, remain very important drivers of drug resistance as they can affect some of the other factors [24]. In addition, unlike most of the other causal factors to anti-malarial drug resistance, drug use practices can be controlled through changes in human behaviours and activities [25]. In order to encourage changes in human behaviours around the use of anti-malarial drugs, there is need for a good understanding of the factors that play important roles in determining these behaviours, and how they interact with one another in influencing these behaviours.

Evidently, most of the studies on anti-malarial drug resistance have been conducted from parasitological [26–29] and pharmacological [21, 24] perspectives. These studies, while adopting a biomedical approach, have mostly looked at the molecular, biological and/or pharmacokinetic aspects of anti-malarial drug resistance. Although they have contributed to the fight against resistance to anti-malarial drugs by providing evidence on the mechanisms involved, the persistent development and spread of resistance to anti-malarial drugs by the *Plasmodium* parasites despite these efforts calls for additional approaches in studying this important public health issue. In order to have a wider picture of the issue of anti-malarial drug resistance, there is need for a psychosocial perspective that will explore the underlying causes of human behaviours that promote the development and spread of resistance. This will help to protect the efficacy of malaria treatments by informing effective behavioural change campaigns. Currently, there is no existing qualitative enquiry that focused specifically on exploring the interactions between socioeconomic factors, anti-malarial drug use behaviours and drug resistance.

Consequently, a social epidemiological stance was adopted in this study. As a sub-discipline of epidemiology, social epidemiology is a perspective on health that is concerned with the connection between social systems and population health [30]. It deals with the social distribution and determinants of states of health [31]. By adopting a psychosocial approach to health, social epidemiologists see health outcomes as not necessarily products of biological factors alone, but also of socio-structural forces acting around individuals [32]. In addition, evidences from existing studies have shown that health-related behaviours are not randomly distributed in nature but are socially and economically patterned [31]. In malaria treatment-seeking, socioeconomic structures can constrain choices of where to seek treatment, affect knowledge level and perception about malaria, and encourage behaviours that might be formed as coping strategies to cushion the effects of these forces. This study explored the underlying socioeconomic factors that determine drug use behaviours promoting anti-malarial drug resistance.

## Research aim

The aim of this study is to explore the anti-malarial drug use behaviours of people from different socioeconomic backgrounds.

## Methods

### Study setting

**Fig. 1:**
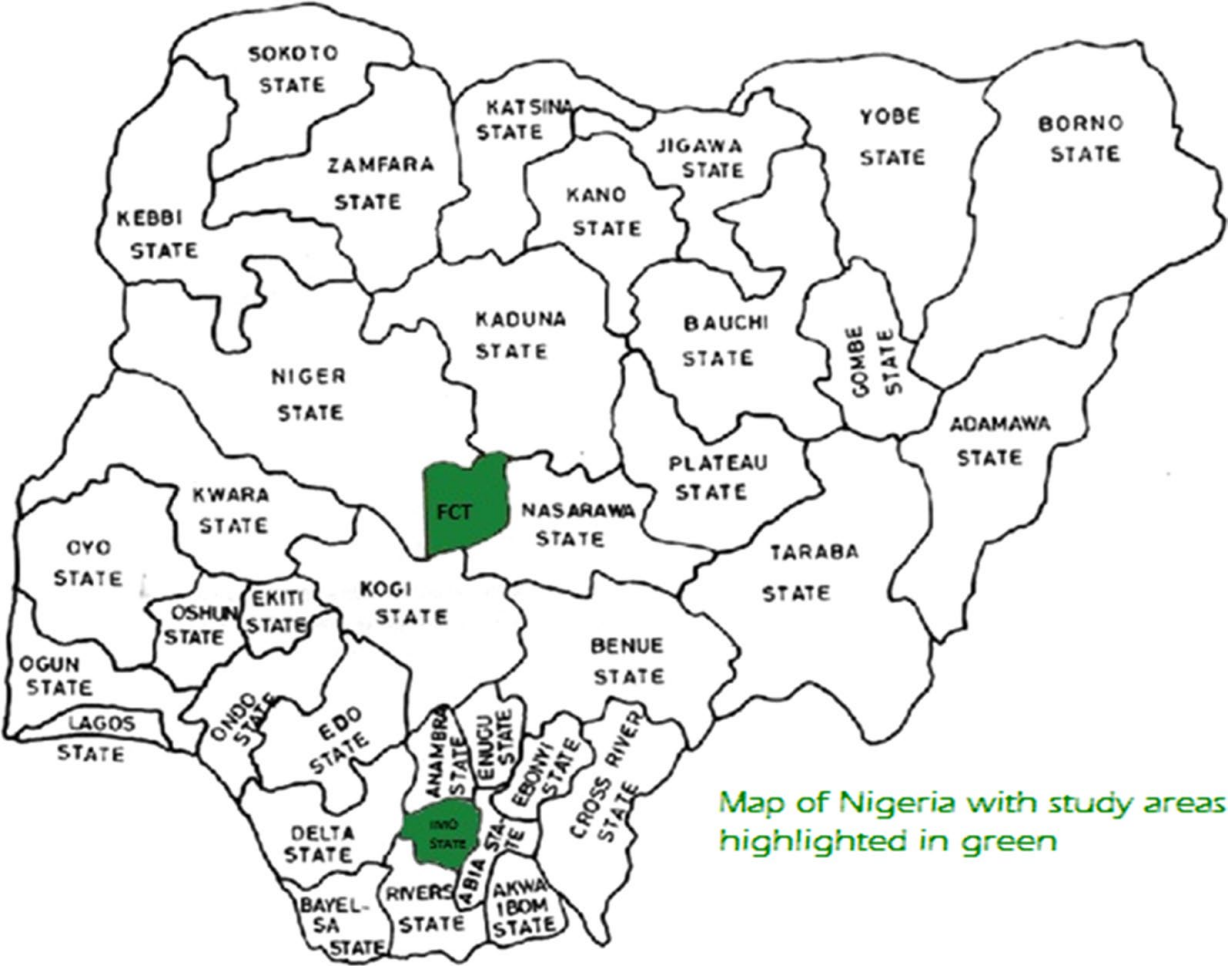
Map of Nigeria with study areas highlighted in *green*

This study was conducted in two areas, Imo state and Abuja in the southern and northern parts of Nigeria, respectively (see Fig. 1 showing a map of Nigeria with the study areas highlighted in green). The interviewees were selected from a rural and an urban area from each of the states, making a total of four (4) study sites—Abuja municipal (Urban); Zuba (rural); Owerri municipal (urban) and Ihitte-uboma (Rural).

Nigeria is a sub-Saharan African country with a population of about 177 million people [33], and about 53% of the population living in the rural areas [34]. The country has a land mass of 923,768 square kilometres and a tropical climate with two seasons: the wet and the dry seasons. The annual rainfall ranges between 550 mm in some part of the north (mainly in the fringes of Sahara desert) to 4000 mm (in the coastal region around Niger delta area in the south). The temperature in Nigeria oscillates between 25 and 40 °C.

The climatic and geographic features of Nigeria make it a conducive site for high malaria transmission. All the states in Nigeria are holoendemic for malaria, and transmission is usually year round. However, the wet or rainy season is the peak malaria transmission season in Nigeria. In addition, the duration of the peak transmission season differs in different locations with the areas in the north having shorter duration of transmission (approximately 3 months) than the south (almost year round) [5]. *Plasmodium falciparum* is the most prevalent (97%) malaria parasite specie in Nigeria [5].

Apart from the geographical and climate change differences between the northern and southern Nigeria; as with most countries in the world, like England, the north–south divide exists in Nigeria with regards to predominant cultures, religion, educational level, type of occupation and level of urbanization. As such, to get a northern and southern perspective of the issue under study, Imo state in the south and Abuja in the north were selected for this study. Other factors, like participants and researchers’ health and safety, were considered in choosing these sites.

### Study design

A qualitative approach, involving the use of interviews, was used in this inquiry to explore the existing anti-malarial drug use practices in the Nigerian population; and the different socioeconomic factors influencing the behaviours. This study is part of a larger study on anti-malarial drug resistance in Nigeria. Fifteen in-depth, face-to-face interviews were conducted with household heads (thirteen), a drug vendor and a pharmacy assistant from the four locations this study covered.

### Sampling and recruitment

The inclusion criteria for participants in this study were:

- Participants must be at least 18 years of age.
- Participants must be resident in Nigeria.
- Participants must be household heads who bear the financial cost of malaria treatment for themselves and members of their household. The reason for this is to ensure that those who can provide accurate information on the financial cost of malaria treatment to their household are recruited.
  In addition to the above, a drug vendor and a pharmacy assistant from the study sites were interviewed to help interpret the findings from the participants.

To ensure that participants who can recall and provide relevant information to answer the research question were selected, a systematic theoretical sampling technique [35] was used in selecting participants for this study. This technique involved continuous sampling and data collection until saturation was achieved; that is, the point when no new information or category, in relation to the issue under study, was emerging from subsequent interviews [36]. Consequently, the data collection and analysis were simultaneously conducted with each interview transcribed and analysed prior to the conduct of the next interview. With this strategy, emerging codes were considered and incorporated into subsequent interviews. This enabled the researcher to probe or elicit further explanations on some behaviours or experiences identified in previous interviews. This strategy was also important in determining when saturation was achieved in this study. In all, fifteen (15) individual in-depth interviews were conducted with fifteen participants.

Prior to the conduct of each interview, the interviewer made a brief first contact with each participant as an ice-breaker strategy to improve acceptance, trust and communication. It was during these first meetings that the participant information sheet was given to the participants and verbally explained. Also, during this contact, the researcher made some observations like the willingness or reluctance of the individuals to participate in the study with the aim of excluding those who show reluctance and are uncomfortable all through the meeting. All individuals approached agreed to this first meeting and were included in this study afterwards as they all met these criteria. Subsequently, the interviewer and the participants agreed on a convenient day, time and venue for the interview.

A written informed consent was obtained from participants prior to the interview. In addition to this, a confirmatory verbal informed consent was obtained and audio recorded at the beginning of each interview. All participants consented to the interview being audio recorded.

Confidentiality and anonymity were assured to all participants. These were maintained all through the study by assigning identification codes to each participant and by storing the interview data securely in a password protected storage device. Prior to the commencement of each interview, participants were informed of their right to partake or not, and to withdraw or ask the interviewer to stop the interview at any point. Participants were also given the contact detail of the researchers and the University of Sunderland Research Ethics Committee for any subsequent concerns. A 1-week period from the day of the interview was given for the participants to contact the researchers if they decide to withdraw their interview account from the study.

The interviews were conducted by the first author whose familiarity of the study population and previous research experiences in Nigeria were very important in the recruitment and conduct of the interviews. The first author, having lived in Nigeria for more than 20 years, had a grounded knowledge and understanding of the local languages and culture of the study population.

Furthermore, the interview questions were centred on participants’ experiences and behaviours in using anti-malarial drugs. Prior to the main interview, the interview schedule was piloted with two participants of similar characteristics with the main participants. The interviews were held at community venues, which participants selected and were already familiar with. In all, the interview time was between 30–35 min. Interviews were conducted in English and two Nigerian languages (Hausa and Igbo languages). Socio-demographic data were collected using a brief questionnaire administered to each participant at the end of the interview.

### Data processing and analysis

All the interview records were transcribed verbatim from audio to a written form by the interviewer on the same day they were conducted. This helped to ensure that the memory and observations during the interviews were retained. Where necessary, the interview transcripts were translated from local language to English.

The thematic analysis method, as outlined by Braun and Clarke [37] was used in analysing the interview data. This involved firstly, a comprehensive reading and re-reading of all the data set (transcript). In the second step, relevant pieces and expressions from the transcript were labelled or coded. Coding, in this study, was conducted at a descriptive level with the aim of summarizing the main concept of each statement from the interview thereby retaining the information from the statement. The third step involved further review of the codes for patterns and linking related codes together to form categories. The grouping into categories was based on not just exact or close similarities; but codes that had something in common or are linked in some ways, that might not seem so at the face value, were also grouped together as a category [38]. The final step was about developing key themes from the categories. The themes were developed around reported practices and behaviours in seeking malaria treatment that were connected. To achieve this, the categories were considered for relevance and how they connect and interact with one another. Categories that were similar or informed others were combined to build up a key theme. For the meaning of some key terms used in this study, see Table 1.

**Table 1:** Key terms used and their meaning in the context of this study

Terms	Meanings
Pharmacy	Used to refer to the registered pharmacy shops that are usually found in the cities. These shops are usually owned or run by a trained pharmacist. Some pharmacies in Nigeria are however run by pharmacy attendants or nurses
Chemist/drug vendors	Used to refer to drug vendors or patent medicine vendors in the study setting. These are usually traders with little or no pharmaceutical trainings who either hawk or sell drugs in a small shop. They are mostly common in the rural and suburban areas in Nigeria. The term chemist and drug vendors were used interchangeably in this study

### Socio-demographic description of participants

The socio-demographic characteristics of the participants are shown in Table 2. Overall, 53.3% of the participants were females. The mean age of the participants was 40 years. The most common occupation of the participants was farming. Almost half (46.7%) of the participants reported they earn below the current Nigerian minimum wage of eighteen thousand Naira (₦18,000) (about £64) per month. Seventy three percent (73%) of the participants had at least some secondary education. Urban dwellers constituted 53.3% of the participants, see Table 2.

**Table 2:** Socio-demographic description of participants

Characteristics	Number (%)
Total no. of participants	15 (100)
Household heads [13]	
Drug vendor [1]	
Pharmacy attendant [1]	
Sex	
Males	7 (46.7)
Females	8 (53.3)
Age	
Mean age	40
Mode age	31
Relationship status	
Single	3 (20)
Married	6 (40)
Widowed	6 (40)
Educational level	
No formal education	1 (6.7)
Some primary school education	3 (20)
Some secondary school education	2 (13.3)
O’level/SSCE holder	2 (13.3)
Tertiary education/degree	4 (26.7)
Post graduate degree	3 (20)
Income level	
Below ₦18,000 (£63)	7 (46.7)
₦18,000 to ₦50,000 (£64 to £177)	1 (6.7)
₦50,001 to ₦100,000 (£178 to £355)	2 (13.3)
₦100,001 to ₦300,000 (£356 to £1065)	3 (20)
Above ₦300,000 (£1065)	2 (13.3)
Type of settlement	
Urban	7 (46.7)
Owerri in Imo state [2]	
Abuja Municipal city [5]	
Rural	8 (53.3)
Nkwumeato community in Imo state [4]	
Zuba community in Abuja [4]	

### Issues of validity and reliability

Lincoln and Guba [39] argued that approaches to the validity and reliability of quantitative research were not appropriate for qualitative studies and they suggested credibility, transferability, dependability, and confirmability as more appropriate criteria. Credibly is ensuring that the findings of a qualitative study are credible and actually reflect the situation under investigation. Merriam and Tisdell [40] posed the question “How congruent are the findings with reality?”. This was addressed through the use of the respondent’s own words to back up claims and by ensuring the outline of the categories was reflective of the interview transcripts. Shenton [41] also suggests that a wider range of respondents can add to the credibility of the study. Respondents were chosen from a range of socio-economic groups and from rural and urban settings. A range of views was, therefore, represented and differences and similarities among the respondents were highlighted in the report. Another approach was validation of the data from the participants themselves (by summarizing their accounts during the interview and asking them for a validation of this) and from other participants (through constant comparative method to validate the experiences of other participants). Furthermore, the interviewing researcher’s familiarity with the study setting was very important in establishing a good communication with the participants, and understanding the social context of the study population—language, culture, social structure, and healthcare system.

Dependability refers to the internal consistency. It is recognized that in qualitative research, the theoretical position of the researcher can affect the direction of the analysis. This factor is recognized, but this study was an exploration of relatively concrete behaviour and the dependability largely was achieved through the quality of the data analysis. Thematic analysis [37] was used as the method of analysis and a clear process was followed, open to scrutiny. The process of analysis was carried out by one of the researchers and then reviewed by the other researchers. All researchers were involved in the discussions and decisions in the analysis of the data—for example, in deciding which categories constitute a key theme from the analysis.

Indeed, confirmability, which refers to the objectivity of the study, can be complex in qualitative research as the researcher will bring his or her particular theoretical position. The key issue here is whether the data analysis was logical and consistent, and if the researchers were transparent in all processes; this was a guiding principle in the study design. Transferability equates to external validity, and again in qualitative research this can be difficult as samples are small and often not representative. Generalization is about: can the findings be transferred to another setting or is the presentation of the data clear enough to allow the reader to do this? The sample was small and examined inhabitants of only two Nigerian areas, however it did identify strategies and behaviours which allow comparison and in this sense transferability was achieved.

## Results

The social structures existing within societies and their socioeconomic indicators are usually based on characteristics that are valued in the society—educational level, income level, type of settlement, occupation, employment status, among others. As indicators of socio-structural position within the society, an individual’s level of socioeconomic measures can determine the likelihood of healthy or unhealthy exposures, behaviours and outcomes [42].

Several key themes emerged from the analysis of the interview data. The themes were developed around the anti-malarial drug use behaviours reported, and their associated socioeconomic factors. The socioeconomic factors identified in this study—income level, type of settlement, educational level and occupational/job type- are all related to one another with some (for example, educational and income levels) acting as determinants to the others. The significant malaria treatment behaviours influenced by socioeconomic factors in this study were ‘mixing’, presumptive treatment of malaria, the use of drug vendors, sharing of drugs, and the use of anti-malarial monotherapies.

The Donabedian model, which was initially developed to serve as an evaluative framework for quality of healthcare [43], has been widely used in studying other aspects of healthcare [44]. It assumes three main components in studying health outcomes: Structure, Process and Outcomes [45–47]. These three domains work in unidirectional way with each domain influencing the next. This was an appropriate way of organising the categories established through the analysis of the interviews.

**Fig. 2:**
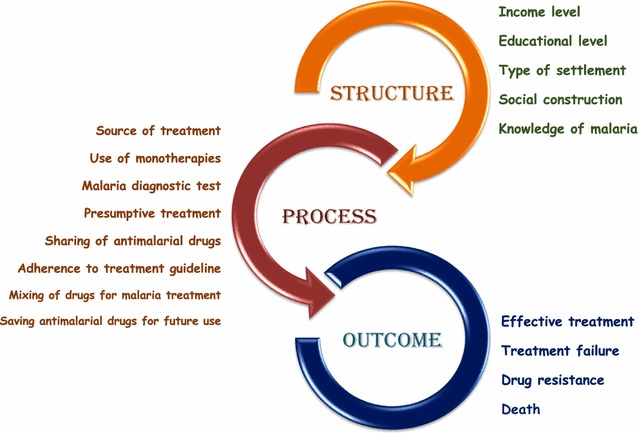
Adapted Donabedian model showing study findings

In applying the Donabedian model to this study (see Fig. 2 of an adapted Donabedian model showing study findings), the ‘structure’ represents all the factors that affect the context in which malarial treatment is sought. These factors control how patients and providers act in seeking and providing malaria treatment respectively. This includes background characteristics of the patient like socioeconomic factors (measures of educational level, level of income and wealth, type of settlement and occupational type) as well as socio-cultural factors (like beliefs, norms, and constructions). The structures in this study were identified from the participants’ interview accounts as well as the socio-demographic questionnaires completed by all the participants. These structural factors influence the ‘process’.

The ‘process’ represents practices and behaviours of the participants in treating malaria, which are products of the interaction with the structures. This includes reported behaviours like drug ‘mixing’ for malaria treatment, presumptive treatment, non-adherence to treatment guideline, use of monotherapies, among others. These behaviours were influenced by the structural factors, which have the ability to limit people’s choices, how they seek malaria treatment and how they use anti-malarial drugs.

Importantly, the process subsequently determines the ‘outcome’ which can be in the form of treatment failure, drug resistance, effective treatment or death. Most of the behaviours identified through the interviews that constitute the ‘process’ have the tendency to encourage the development and/or spread of anti-malarial drug resistance.

## Structure

### Socioeconomic factors

As earlier stated, socioeconomic factors like income level, type of settlement and educational levels constitute structures that can enhance or endanger a population’s health. These factors were reported to affect the way malaria treatment is sought and how anti-malarial drugs are used. For instance, educational level affects one’s ability to read and understand written instructions on how to administer anti-malarial drugs.

*‘Yes. But even if they write it, did I go to school? What I know is that way they have shown me to take it, that’s the way I follow’* (Female, rural area).

*‘Let me say the poor condition that some of us we are living in because we don’t have access to eh this eh hospitals* –*the so called hospitals they are talking about because we don’t have money’* (Male, 32 years old, rural area).

### Knowledge and perception on malaria

A key issue was the level of knowledge of malaria, which influences preventive and treatment seeking behaviours. Some of the perceptions about cause of malaria demonstrated by the participants (like the perception that consumption of fatty diets as well as spiritual attacks as a cause of malaria) were products of social construction from the different communities. All the participants identified the link between mosquitoes and malaria and they also were aware of some indirect causal factors, such as: dirty environment, living in dilapidated housing condition, stagnant waters around the house, among others. However, these indirect causal factors were perceived as direct causes of malaria by many of the participants.

*‘What I know about malaria is that, some people say taking too much of oil* -*oil is one of the causes of malaria, palm oil. Another thing I know that causes it is like, for example, some people might be asked not to eat something like ‘egusi’, ‘egusi’ causes malaria too’* (Female, 49 years old, rural area).

As malaria is a common disease, which all the participants had experienced, knowledge of malaria symptoms was satisfactory among the participants. There were, however, some cultural and superstitious constructs and explanations for malaria infection; three participants said they believed some evil spiritual powers can cause malaria. This perception was reported in relation to the cause of malaria as well as the manifestation of the disease:

*‘Some people say it’s a spiritual attack. You understand. Because it was very mysterious* -*my mouth was very bitter but the symptoms were malaria symptoms. I don’t know if anything passed through the malaria’* (Female, 33 years old, urban area).

At one end of the continuum many of the participants saw malaria infection as life threatening; however, others saw it as a very common disease which is easy to treat hence not a life-threatening or serious infection.

*‘Well, maybe because I am a Nigerian, I don’t see it as a life threatening disease. But I know that eh, when you travel abroad, they don’t want* *to even hear that you have malaria. Malaria to us or to me personally, is like having headache. Am serious about that’* (Female, 40 years old, urban area).

## Process

### Malaria treatment-seeking behaviours

When symptoms were experienced, socio-economic factors, like type of settlement, income level and type of job, tended to determine the treatment behaviours and, therefore, informed and determined the experience of the illness. This was in terms of first point of contact for treatment and choice of anti-malarial drugs; as these involved money, socioeconomic status was a determinant. Conversely, other behaviours, like presumptive treatment, were reported across all socioeconomic groups in this study.

On experiencing malaria like symptoms, there are several facilities one can seek treatment in Nigeria: hospitals/clinics, health centres, chemists/drug vendors, pharmacies and herbal healers. These facilities usually offer different treatment options ranging from a laboratory diagnostic test prior, to presumptive treatment based on presenting symptoms. Those living in urban areas tended to use the pharmacy or hospital as the first point of contact whereas in the rural areas pharmacies and hospitals were less common as “chemists” were easier to access hence more used. With a large proportion (53%) of the Nigerian population living in rural areas, the chemists/drug vendors serves as the first point of contact for malaria treatment for many. The pharmacies provide professional and more expensive services whereas the drug vendors often lack the knowledge and skill which was reflected on both advice and cost of services in these facilities. The chemist/drug vendor was the most reported first point of seeking malaria treatment among participants whose monthly income was below the ₦18,000 (£63) minimum wage. Even when other sources were closely located, the chemists/drug vendors remained the most commonly used source for malaria treatment by those of low socioeconomic status. The seeming trust in the chemist/drug vendors by the rural dwellers was not based on their ability to offer more effective treatment than the hospitals, health centre or pharmacies. Rather, it was because, unlike the other sources, the chemist/drug vendors are more available and accessible through the provision of credit facility, cheaper alternatives, and convenient locations and out-of-hours services.

*‘… hospital is not far from me, from where I leave… why I don’t go there is that those two chemists, they ‘help’ me…. That’s why I go to theirs. So if I don’t have money they help me. If it’s the government hospital, everybody comes from different places to work. They will say pay us before we give you the drugs’* (Female, 31 years old, rural area).

The participants were fully aware of the difference in standard between the pharmacies and the chemists, but both availability and low cost determined the use of the chemists.

*‘The way we look at chemist in Nigeria is that chemist is meant for, you know, people that are in the villages, you know that cannot afford (laughs) because in that chemist you see all sort of, manner of drugs… you see fake drugs there’* (Female, 44 years old, urban area).

Furthermore, when symptoms were severe or when seeking malaria treatment for children, most of the participants would use hospital or pharmacies as the first point of treatment.

### Presumptive diagnosis and treatment of malaria

Presumptive treatment of malaria, which involves treating suspected malaria cases based on presenting symptoms rather than a confirmatory diagnostic test [48], was the commonest approach and was found in all socioeconomic groups. Those who mostly use the hospital for treatment, who were also the highest earners with the highest educational levels, reported the use of laboratory testing to confirm diagnosis more than those who use the chemist/drug vendors or pharmacies. The pharmacies tended to implement treatment on the basis of presumptive diagnosis; and chemist/drug vendors almost entirely implemented treatment on the basis of presumptive diagnosis.

An example of this is with the three participants who reported they have never gone for a malaria diagnostic test before (neither microscopy laboratory nor RDT) as they all used the chemist for most malaria treatments. The most common reasons given by these participants for this practice were their inability to afford the treatment cost, lack of access to hospitals and cultural factors. These three participants were rural dwellers and earned below the minimum wage of ₦18,000 (£63) per month.

*‘…I don’t know whether some of the sicknesses I experience sometimes are malaria. I may* –*I might have had it, but I may not know it was malaria* –*I think I have told you that I have never gone for any malaria lab test to test me, to justify what is really my problem’* (Male, 32 years old, rural area).

Another reported behaviour with regards to presumptive treatment and the use of malaria diagnostic test prior to treatment was the practice of using previous results of malaria diagnostic test and the prescription received to treat subsequent episodes with similar symptoms. This was reported by two participants from the rural areas.

*‘… sometimes, I go to the health centre. There was a time I went there and they ran a test and said its malaria and typhoid. They treated and gave me some drugs and also directed me to nearby pharmacy to buy more drugs and add to the ones they gave me and take them all. After the treatment, when next I have malaria, I go to the pharmacy and buy the same drugs. Its not all the times that I have the money to run a test’* (Male, 55 years old, rural area).

In all, only one participant said she always used a diagnostic test to confirm she has malaria prior to her use of anti-malarial drugs. The other participants who also use laboratory diagnostic test prior to malaria treatment were not consistent as they also reported they practiced presumptive treatment. These were mostly participants who earned more than ₦100,000 per month. For the high-income earners in this study who practice presumptive treatment, affordability was not reported as a reason for this behaviour. The main reasons for this practice include: confident they can diagnose malaria and the perception of presumptive treatment as time-saving route to malaria treatment.

Similarly, affordability was not always the key issue in decisions on the type of anti-malarial drug to get among different socioeconomic groups. The pharmacy attendant reported that most of the customers at the urban area are not really concerned about price but on the effectiveness of the drug, hence ACT was reported as the most sold class of anti-malarial drugs by the pharmacy attendant. This was however different in the rural areas and the low socio-economic groups; as would be expected, affordability was an important issue for these groups. The cost of ACT was reported as out of reach by many rural and urban dwellers who earned below the national minimum wage. As a result, monotherapies and non-ACT, like SP (which are cheaper and less effective in parasite clearance), were reportedly the most used anti-malarial drugs by low-income earners and rural dwellers.

*‘Like if severe, most of the people do not have enough money to treat themselves. They may tell you they need drugs of two hundred naira or one hundred and fifty naira. At times I have to use Laridox (sulfadoxine*-*pyrimethamine)’* (Male Drug Vendor, rural area).

### Adherence to treatment guideline

As an important factor in the development of drug resistance, adherence (in terms of completion of treatment course and administration of the proper dose at the correct time, in accordance with the WHO malaria treatment guidelines [49] was considered in this study. Many participants reported non-adherence at least once in the last 6 months. Generally, reports of poor adherence were more common among participants of lower level of education and income level of below ₦50,000 (£177). However, some low-income participants, who reported a moderate level of adherence, saw anti-malarial drugs as expensive, so when they do buy them, they tend to complete the treatment as they do not want to waste their (expensive) treatment, and so wants value for their money.

*‘There are times they will give me and I will take it and take it and take it, if the thing (malaria) clears, I will throw away the remaining. Even if its injection, I will not go to complete it. Because the thing (malaria) has cleared’* (Male, 55 years old, rural area).

*‘Yes. If they give me* *drugs, I finish them. I don’t leave them because I used my money to buy them. Any drug they give me in the hospital I must take all of them. I don’t miss it. I don’t miss it as* -*are my not the one that will bear the pains? How can I miss it? If I don’t take it I will feel the sign in my body of course’* (Female, 35 years old, rural area).

While ideas of cost effectiveness encouraged adherence in some low income earners, less commonly (mentioned only by two participants), it contributed to some non-adherence practices like stopping the treatment to save the drug for future use. The practice of stopping to save drugs for future use was not common as most of the participants were wary of this practice as it was seen as risky and there was a fear of taking expired drugs.

*‘When I buy and give him and he takes and gets better and says he will not take anymore, I throw the rest away. Because when you keep it, do you know the day the sickness will come? By the time the sickness come, and u give it to him, it might bring another sickness for him because you don’t know if the drug has expired’* (Female, 31 years old, rural area).

Sharing of anti-malarial course among two or more people was also reported. One of the three participants who reported this behaviour was of high income (above ₦300,000 (£1065) per month) and high educational level (postgraduate education). The difference between this participant’s behaviour and that of the other two (of income level below ₦18,000 (£63) and some primary school education) is that for the former, it was a one-off act because she could not buy a new course for a member of her household who got ill in the middle of the night; while for the later, it was reported as a recurrent behaviour. In most cases of sharing anti-malarial drugs with others, all parties involved end up not getting a complete treatment course.

### Use of monotherapies

Generally, the knowledge of the names of anti-malarial drugs used was poor among the participants. Nevertheless, most of those who could identify the names of the anti-malarial drugs they use reported artesunate as the most used anti-malarial drug, (five from the rural area reported using this drug compared to two from urban sites). Additionally, the drug vendor and pharmacy shop assistant that were interviewed also reported the sale of monotherapies (like artesunate) and non-artemisinin based combinations (like sulfadoxine-pyrimethamine (SP). The drug vendor in the rural area described a higher rate of monotherapy sale than the pharmacy attendant in the urban area. The major reason given for this by the drug vendor was the inability of the customers in the rural areas to afford the price for the artemisinin-based combination therapy, which is the first-line treatment for malaria in Nigeria.

### Mixing

The concept and practice of ‘mixing’ was first mentioned in the initial interviews with participants from Zuba (rural area) in Abuja, and then subsequently in most of the interviews from other rural areas. To clearly understand the practice of mixing and how it is done, a drug vendor from one of the communities in the rural areas and a pharmacy attendant from an urban area were interviewed. They described mixing as the practice of combining different drugs to treat malaria. It is done by including, for example, a dose from each of the included drugs to form a mixed dose. There is no standard in terms of the number or types of drugs used in the mixture for malaria treatment.

*‘Almost all the people here normally mix drugs. They may come and say I need a malaria drug of fifty naira or hundred naira. … I don’t have much money to buy a complete one’* (Male Drug Vendor, from rural area).

Mixing was described as a very handy and flexible coping strategy by the low income earners. Also, all the participants from the rural areas reported they mixed. The highest level of education attained by most of those who mixed was some primary education.

*‘No I haven’t taken those packaged as complete course. The ones they mix, that’s the ones they buy for me. That’s the one our hand can get to. We only buy the ones we can afford’* (Female, age not known, rural area).

*‘If you have up to five hundred naira or more, we can even mix Artesunate or Paracetamol or any analgestic, Panadol, Amoxil or Septrin’* (Male Drug Vendor, from rural area).

The anti-malarial drugs bought from the pharmacies were mostly sold as a complete course in their original packets. As the mixed drugs are sold in units of ‘one mixed dose’, a very important factor in determining how many doses of the mixture a customer gets is the amount they can afford. The customers can decide to buy a day’s dose or 2 days’ dose and so on. While the complete course of anti-malarial drugs like artemether-lumefantrine was reported to be about ₦800 (£2.84); ₦100 (£0.35 p) will get you a day dose of mixed anti-malarial drugs if you cannot afford ₦800. Another important issue on the ‘mixing’ practice is that these drugs are not sold in their original packets as they are either cut up into bits or removed from the blister pack while being dispensed. Hence the customers/patients have little or no information about the individual drugs that constitute the mixture nor their expiry dates.

*‘The last time I used anti*-*malarial drugs, I bought it from the chemist. They mixed the drugs for me. They mixed that of two days. … how they mix is, it depends on how many days’ dose you want them to mix for you’* (Male, 55 years old, rural area).

## Outcome

As earlier stated there are four likely outcomes from the reported practices and behaviours indicated under the ‘process’. These include recovery—which can be an indicator of an effective malaria treatment; treatment failure—in the form of lack of response or worsening of the clinical manifestation of the infection after administering an anti-malarial course; resistance to anti-malarial drugs by the *Plasmodium* parasite; or death.

Some participants in this study discussed the outcomes of their malaria treatment experiences. Through the interview data, outcomes like death could not be explored since most participants were interviewed based on their own malaria treatment seeking behaviour. Although drug resistance was not directly measured/reported in this study, it is worth mentioning that malaria treatment failure can be an indicator of drug resistance [24].

Participants’ experiences of the outcomes were different depending on reported practices and behaviours like the type of anti-malarial drug used, adherence to recommendation on how to administer the drugs, amongst others. On their last malaria treatment experience, treatment failure was reported by three participants who used the mixed anti-malarial drugs. Only one of the participants who used a formal health facility during her last malaria episode reported treatment failure.

*“… you just go to the chemist. Because the way I used to even treat my own, when it happens like that, there’s one chemist that is near our house, I will meet the woman and she will just mix drugs and give me. When she gives me I will just give them. They will take. Their body will be ok. When it is ok for like two days, before three days again, they will still complain again. Before you know it, you carry they to the hospital”* (Female, 31 years old, rural area).

*“The difficulty I encounter sometimes is that there are sometimes that malaria will hold me down, and if I go and tell the chemists that malaria is disturbing me, they will mix medicine for me and tell me how I will take them. Even after taking them, it will be like I didn’t take any medicine”* (Male, 55 years old, rural area).

Nevertheless, on their last malaria episode, recovery or effective treatment was mostly reported in line with the practices of seeking treatment from a formal health facility and adhering to the recommended dose and completing the course. As the formal health facilities was reportedly used last time by mostly those from urban areas, many of those who reported effective treatment, without prior failure, were urban dwellers. However, one participant from rural area who used a hospital last time reported an outcome of effective treatment with no prior treatment failure.

*“so I rushed to meet that doctor… he said its malaria. … he started giving me treatment. I didn’t even go to the farm for one week. I stayed home and took all the drugs when I took all the treatments I got myself”* (Female, 31 years old, rural area).

As there are no detailed anti-malarial drug resistance surveillance programmes in Nigeria, some of the reported cases of treatment failure, which can be drug resistance cases, go unreported and not recorded or followed up. The lack of adequate care and surveillance for this potential cases of drug resistance can encourage further spread of resistant *Plasmodium* parasites in the population.

## Discussion

The results of this study indicate that socioeconomic factors play important role in determining anti-malarial drug use behaviours that promote drug resistance. To explicitly interpret the data on the malaria treatment experiences and behaviours of the participants, and to create a holistic insight on how these contribute to anti-malarial drug resistance, the Donabedian model was adapted and used in this inquiry (see Fig. 2). The structure, mainly but not exclusively socioeconomic factors, determined the treatment seeking and drug use behaviours (process), which could in turn determine the outcome.

Evidently, social and economic contexts can influence behaviours as they contribute in shaping norms and in creating opportunities that promote certain behaviours [31]. Indeed, physical, social and economic environments can place constraints on an individual’s choices as well as that of a population. As shown in this study, income level and type of settlement, as structural factors, affect the decision on where to seek malaria treatment and whether or not a malaria diagnostic test will be used prior to treatment. In line with existing malaria studies in the Nigerian population [8, 50, 51], the chemist/drug vendors and pharmacy were the major sources of malaria treatment. While some level of good practice and quality of care exists in the pharmacies which are usually owned or managed by a trained pharmacist or health practitioner, the quality of care and practice in the chemists, which are owned and managed mostly by traders with little or no pharmaceutical training, remains a big public health concern in Nigeria. The chemists, which are the cheapest and most used source for malaria treatment in Nigeria are also the most likely place to get counterfeit anti-malarial drugs [52, 53].

Previous studies have shown that the cost of the original drugs will influence the circulation of counterfeit anti-malarial drugs [54–56]. These counterfeit anti-malarial drugs are usually sold cheaper than the original ones; hence those in the low socioeconomic gradient constitute the target market for the counterfeiters. As long as ACT remains unaffordable to most Nigerians, the chemist will remain the main source of malaria treatment, especially for the poor, and the circulation of fake anti-malarial drugs will flourish.

The study by Onwujekwe et al. [53] on the quality of anti-malarial drugs provided by public and private healthcare providers in Southeast Nigeria found that 37% of the anti-malarial drugs tested did not meet the United States Pharmacopoeia (USP) specifications for the amount of active ingredients as they either lacked the active drug ingredient or contained sub-therapeutic quantities of it. Populations, like the Pailin province of Cambodia in SEA where artemisinin resistance have been confirmed have some similarity with the Nigerian population in terms of the existence of widely available cheap counterfeit anti-malarial drugs [57].

The public health danger of the sale of counterfeited drugs is that some of them contain inadequate quantities of the active ingredient which can encourage the development and spread of drug resistance by exposing the *Plasmodium* parasites to sub-therapeutic doses [58]. This creates a serious threat to vulnerable populations and jeopardises the progress and investments in malaria control [21]. Modelling analyses have also demonstrated that administration of under-dose of artemisinin plays an important part in the spread of artemisinin resistance [59].

In addition, the practice of mixing will have similar outcome in terms of encouraging the development and spread of anti-malarial drug resistance. With mixing, the patients can buy less or more number of doses than recommended. One of the dangers of using the mixed anti-malarial drugs is that it offers a safe route for the sale of expired and fake anti-malarial drugs as the mixed drugs are not sold or dispensed in their original packets. Another danger from this practice is the possibility that the interaction of the different drugs used in the mixture can alter the efficacy of these drugs when administered.

Furthermore, the low prevalence in this study, of some practices that are known to encourage the development of drug resistance, like sharing of malaria treatment course, stopping treatment to save drugs for future use, among others [60, 61], was not necessarily because of the main dangers associated with these practices. For instance, the practice of stopping treatment to save drugs was low among the participants because those more likely to do this (people of low socioeconomic status) used mostly mixed drugs for malaria treatment as they usually do not know the expiry date of the anti-malarial drugs. Hence, they expressed their fear that saving the drugs might mean taking expired anti-malarial drugs in the future. Also, it is possible that this practice of stopping treatment to save drugs for future use, which promotes the use of incomplete treatment course, could have been higher than reported if the participants had knowledge of the expiry dates of the anti-malarial drugs, especially those that use the mixed anti-malarial drugs.

One of the behaviours that cut across different levels of socioeconomic measures in this study was presumptive treatment. Presumptive treatment has been identified as a major drive to drug over-use which promotes the development and spread of resistance to anti-malarial drugs by the *Plasmodium* parasites [2]. Over-use of anti-malarial drug has been reported to increase the incident of altering within-host ecology of drug resistant parasites; and removing the drug-sensitive competitors has a significant impact on transmission potentials of drug resistant parasites [24].

Finally, the WHO recommended that the use of artemisinin monotherapies for treatment of uncomplicated malaria cases be banned as their use promotes the development of resistance to artemisinin by the *Plasmodium* parasites. Nigeria was actually listed in the latest WHO malaria report [1] as one of the countries that have stopped the use of oral artemisinin monotherapies; however, these drugs (especially Artesunate) are still widely used and readily available in Nigeria and this threatens the efficacy of artemisinin both as monotherapies and in combination with other partner drugs. The wide use of monotherapies is another feature Nigeria shares with some of the countries in the Great Mekong region where artemisinin resistance has been confirmed.

## Limitations of the study

One of the limitations of this study is that it did not further explore the role of socio-cultural factors in determining malaria treatment-seeking and drug use behaviours. Although some socio-cultural factors were identified in relation to malaria treatment, these were not further explored as the main focus of this study was on the contributory role of socioeconomic factors in determining treatment behaviours that promote anti-malarial drug resistance.

Also, this study is limited in terms of generalizability of the findings as it used a small sample and examined inhabitants of only two Nigerian areas. Nevertheless, the diversity of the participants, in terms of socio-demographic characteristics, ensures the transferability of the findings.

## Conclusions and recommendations

Socioeconomic factors are major variables in determining behaviours and practices in malaria treatment. Most of the anti-malarial drug use behaviours that can promote resistance, which were reported in this study, were more among people of lower income level, low level of education, and of rural settlements. Behaviours and practices like mixing drugs for malaria treatment, use of monotherapies, saving anti-malarial drugs for future use, sharing of malaria drug/treatment course, among others are all coping strategies by the less well-off who cannot afford the recommended route to malaria treatment by the WHO.

To achieve a sustainable improvement in health, campaigns should be targeted at social structures in the society that produce diseases [31]. In malaria campaigns, there is need to broaden the scope of anti-malarial drug resistance control strategies to include strategies targeted at improving the socioeconomic status of people in malaria endemic areas. Population-wide improvements in income, education, environmental and structural conditions of rural dwellers in malaria endemic settings, like Nigeria, will encourage behavioural change on how anti-malarial drugs are used.

Governments of malaria endemic countries should ensure existence and enforcement of policies and regulations on anti-malarial drug distribution and dispensing. However, this can only successfully reduce the rate of anti-malarial drug abuse if the existing underlying factors that promote these behaviours, like poverty, poor access to healthcare services, poor level of education, social constructions, are first addressed. There is a need for the Nigerian government to review its policy on payment at the point of use for primary healthcare services as this constitutes a barrier to the use of formal health facilities that provides better quality malaria treatment.
